# Use of a Non-Penetrating Captive Bolt for Euthanasia of Neonate Goats

**DOI:** 10.3390/ani8040058

**Published:** 2018-04-20

**Authors:** Andrew Grist, Jeff A. Lines, Toby G. Knowles, Charles W. Mason, Stephen B. Wotton

**Affiliations:** 1 School of Veterinary Sciences, University of Bristol, Langford House, Langford, Bristol BS40 5DU, UK; toby.knowles@bristol.ac.uk (T.G.K.), steve.wotton@bristol.ac.uk (S.B.W.); 2 Silsoe Livestock Systems, Wrest Park, Silsoe, Bedford MK45 4HR, UK; jeff.lines@silsoeresearch.org.uk; 3 Humane Slaughter Association, The Old School, Brewhouse Hill, Wheathampstead, Hertfordshire AL4 8AN, UK; charlie@hsa.org.uk

**Keywords:** animal welfare, euthanasia, livestock, mechanical killing, on-farm killing, neonate goats

## Abstract

**Simple Summary:**

With animal production systems, there is an inevitable need for the stock person to humanely kill new-born (neonate) animals, either due to disease, malformation, or in instances of production efficiency (males born to a milking herd for example). At present, the standard method employed is manual killing with a blunt object or swinging the animal so that its’ head contacts a hard surface such as a wall. Stockpeople do not like performing this action and it also has consequences for the animal in terms of reproducibility and ability of the stockperson. This study examines the use of a mechanical captive bolt device to produce immediate brain death in neonate goats, causing this state of irreversible brain dysfunction before the animal can feel the procedure, or the effects of the procedure. This study found that a device powered by a blank cartridge, containing a specified amount of explosive (1 grain), when applied in a specific position on the head produced immediate brain death in neonate goats. As such, this method is considered to be a reproducible and humane method of euthanasia, as the brain is destroyed before the animal can feel the shot.

**Abstract:**

A non-penetrating captive bolt device, powered by a 1 grain 0.22″ cartridge delivering a calculated kinetic energy of 47 Joules was tested as a euthanasia method on 200 neonate goats (*Capra aegagrus hircus*) of mean dead weight = 4.425 kg (SD (Standard deviation) ± 0.4632), to assess effectiveness and shot position. Evaluation of the method was conducted using behavioural indicators of brain dysfunction followed by post mortem examination of the heads. Once correct shot position had been established, 100% of 158 kids (95% confidence interval 97.5% to 100%) were successfully stunned/killed with a shot positioned on the midline, between the ears, with the chin tucked into the neck. The use of the Accles and Shelvoke CASH Small Animal Tool can therefore be recommended for the euthanasia of neonate goats with a 1 grain cartridge and a specific shooting position.

## 1. Introduction

Modern production methods inevitably lead to farmers and stockpersons having a requirement to humanely kill neonate goats (*Capra aegagrus hircus*) at some point in the production cycle, be it due to production efficiencies (e.g., male kids produced in a dairy herd), disease outbreak, or neonatal issues affecting the viability of the animal. One of the three desirable personal qualities of the stockperson identified by the Farm Animal Welfare Council [1] is empathy and affinity with the stock they rear; this quality means that in general they do not like undertaking euthanasia of livestock unless the animal appears ill and the euthanasia method is perceived as being pain free [2,3,4]. It also has to be considered that manual euthanasia is a skill that has issues in terms of reproducibility, and there is of course concern for the welfare of animals during training to proficiency.

Previous research (United Kingdom Department for Environment, Food and Rural Affairs, MH0116) has demonstrated that a mechanical stun/kill can offer an acceptable method for the humane killing of neonates and that a non-penetrating percussive device shows less variability than penetrating devices. The use of the Accles and Shelvoke CASH Small Animal Tool (CPK200, Birmingham, UK) [5] with a pink coded 1.25-grain cartridge was tested on 80 goat kids and was subjectively assessed as producing a humane stun/kill (MH0116). Research in New Zealand [6,7] has demonstrated that the application of a mechanical percussive blow applied using a TED [8] butane powered, non-penetrating, captive bolt device that develops a kinetic energy (K.E) of approximately 27.8 Joules [7] was effective at producing a humane stun/kill, provided a specific shooting position was employed. As ‘stunning’ is defined in European legislation [9] as any intentionally induced process which causes loss of consciousness and sensibility without pain, including any process resulting in instantaneous death, in this paper we differentiate between recoverable (simple) stunning “loss of consciousness and sensibility without pain” by referring to it as stunning and “any process resulting in instantaneous death” by referring to it as a stun/kill.

The detailed design and operation of the CASH Small Animal Tool is described in another paper [10]. Essentially, the device is a non-penetrating captive bolt that delivers a kinetic energy of 47 Joules using a 1 grain brown coded 0.22″ calibre blank cartridge [10]. The manufacturer informed the research team that the updated device had been proofed to 1 grain rather than the higher powered 1.25 grain pink coded cartridge as used in the previous study.

This paper reports the methods and findings of DEFRA project MH0150 to investigate the effectiveness of the Accles and Shelvoke CASH Small Animal Killer (CPK 200) powered by a brown 1 grain cartridge to produce a humane stun/kill in 200 neonate goat kids that were approximately ≤8 days old. The study was approved by the University of Bristol’s Ethical Committee and carried out under a United Kingdom Home Office Licence (PPL 30/2999), and was conducted after initial testing on cadavers. 

## 2. Materials and Methods

### 2.1. Experimental Animals

The euthanasia method was applied to 200 British Saanen goat kids of mean dead weight = 4.425 kg (SD ± 0.4632). Saanens are the largest of the dairy goat breeds, a horned breed and are cited by the breed society as one of the greatest milk producers. Statistical advice suggests that 100% efficiency can never be absolutely proven. There will always be some small margin for error, however large the study. However, if we demonstrated for example, with 200 animals, we would be 95% confident if 100% of 200 animals were effectively stun/killed that the very maximum percentage of animals not immediately stunned/killed would be never more than 1.9%. Based on a confidence level of 95% a sample size of 200 was recommended as a sensible balance between animal use and demonstrating the degree of efficacy.

The animals used in this study were predominantly male goats that were surplus to the requirement of a dairy herd and are normally killed within their first week of life. Participating producers were instructed that any animal requiring euthanasia on welfare grounds must be immediately and humanely dealt with and not saved for the research trial. Research by Sutherland et al. [7] assessed the effect of anatomical differences between male and female kids and the effectiveness of a non-penetrating captive bolt gun to produce a humane stun/kill. They demonstrated that there was no effect of gender on the effectiveness of the shot or the duration of convulsions produced and in addition, anatomical skull differences in this age of goat are insignificant. Therefore, we can justify the use of male kids to gain a representation of the effect of the percussive blow on both genders.

**Figure 1:**
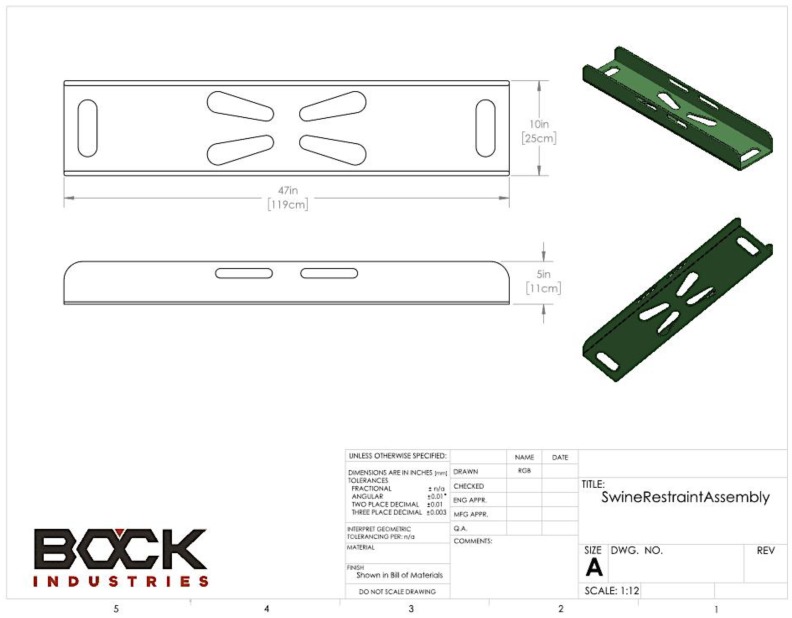
Bock Industries (Philipsburg, PA, USA) restrainer.

**Figure 2:**
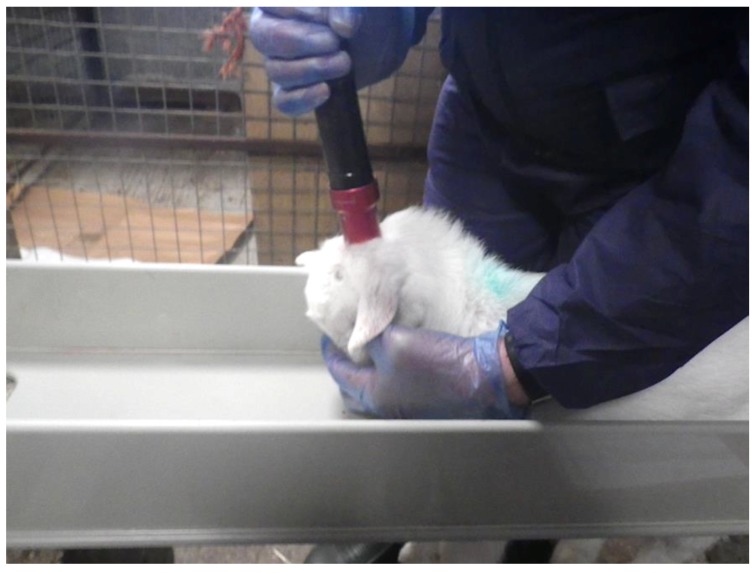
Restrainer in use (revised shot position technique, see below).

The kids were individually restrained within a non-flexible, plastic restrainer that was designed and built by Bock Industries (Philipsburg, PA, USA) in collaboration with the University of Bristol (Figure 1). The use of this restrainer enabled the humane kill to be completed by a single operator, a practical requirement of stockpersons, on-farm. All animals were shot once by an experienced researcher (Stephen B. Wotton) using the CASH Small Animal Tool with a 1 grain brown coded 0.22″ cartridge (Figure 2) whilst the kid was gently held down with the free hand. After shooting, behavioural indicators of brain dysfunction were assessed by the same researcher, together with subjective scoring of post shot movement based on the criteria given in Table 1. The presence of post-stun/kill movement (clonic convulsions) is an expected outcome with effective mechanical stunning, where spinal reflexes are ‘out of the control’ of the higher centres of the brain and are thus exaggerated.

Previous research [10,11] has shown that the following measures allow assessment of the effectiveness of the application, i.e., the absence of:

1. Rhythmic breathing. This is controlled by the medulla in the brainstem and is the most reliable indicator of a stun, and if it does not return, cortical brain death (stun/kill).
2. Positive corneal reflex, a brainstem reflex.
3. Positive palpebral reflex, another brainstem reflex.
4. Response to a painful stimulus (e.g., needle prick to the nose).

Any animal that showed signs of recovery including rhythmic breathing within a three-minute post-application evaluation time or, that persisted beyond the three-minute evaluation time, was killed immediately using a schedule 1 method (injected overdose of the pentobarbitone ‘*Euthatal’* Merial, UK GTIN:03661103015550 1 mL 200 mg/mL injection into the heart).

If the severity of the concussion is sufficient to affect the brainstem the animal is either stunned, dying, or dead and if the absence of the above criteria persists for ≥3 min, the animal cannot recover [12]. Cardiac function following a severe percussive blow may persist for several minutes and is not indicative of continued brain function.

The presence of post-stun/kill movement (clonic convulsions) is an expected outcome with effective mechanical stunning, as previously described. As this post-shot movement may be undesirable for many operatives it was scored from 0 to 3, using the criteria shown in Table 1. Movement scoring and the time from application to the loss of movement was recorded for analysis so that training material can be developed explaining the relationship between a successful stun/kill and subsequent movement to allay any concerns that may arise from the aesthetic issues of post stun convulsions.

After application of the euthanasia method and assessment, each kid was numerically ear tagged with the kill number and placed in a bag before being hard frozen, pending post mortem examination to facilitate correlation between recorded behavioural indicators and post mortem findings. All assessments, and later post mortem findings, were compiled in a Microsoft Excel database for later statistical analysis.

### 2.2. Post Mortem Examination of Heads

**Figure 3:**
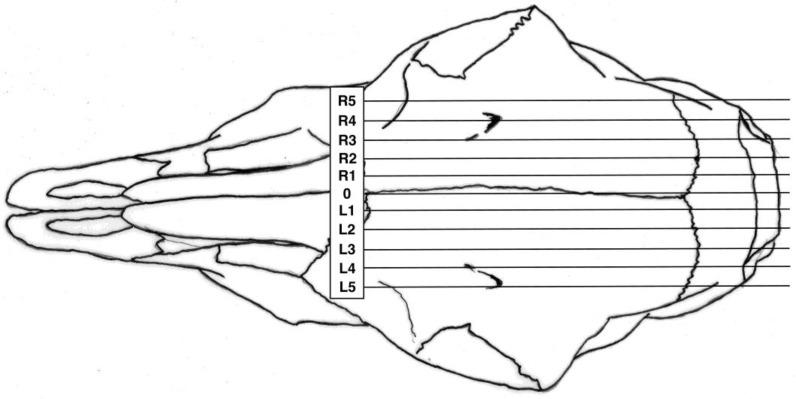
Diagram of lateral target area, with 0 representing the midline based on the suture of the paired frontal bones, L figures being to the left and R figures being to the right of this midline.

**Figure 4:**
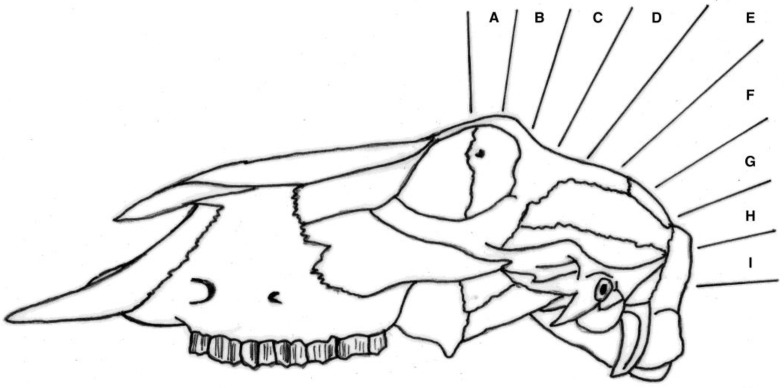
Diagram of sagittal targeting areas using surface topography with F representing a shot on the frontal–parietal suture, G on the parietal bone, H on the parietal–occipital suture etc.

All experimental animals were frozen after killing and subsequently thawed for post mortem examination. The head was examined for external lesions, including laceration at the point of impact. For all carcasses, the skin from the head was removed following a cranial T incision to the shoulders and extending forward to the nose. The impact site was photographed with a digital camera (PENTAX Optio WG-1 or PENTAX K-50 (Ricoh Imaging Europe, Rungis, France)) before removal of any haematoma and the periosteum to expose fracture lines extending from the impact site. Photographs were taken of the fracture patterns to allow for later comparison. The heads were removed from the carcasses and placed in a sequentially numbered bag with the corresponding ear tag and subsequently individually hard frozen to facilitate sectioning on the sagittal plane for photography of cranial and brain lesions to be undertaken. Freezing prior to cutting with a bandsaw prevented distortion of macroscopic lesions. To determine the most effective application position the location of each shot was recorded retrospectively during post mortem based on the lateral and sagittal guide (Figure 3 and Figure 4) to produce a shot code for each head.

Each head was removed from the bag once the number had been noted; the ear tag number was checked to ensure correlation. The head was split on the sagittal plane using an electric band saw (Startrite Meat Master, UK) and both sides were photographed on the medial plane with a digital camera and post mortem findings recorded.

**Figure 5:**
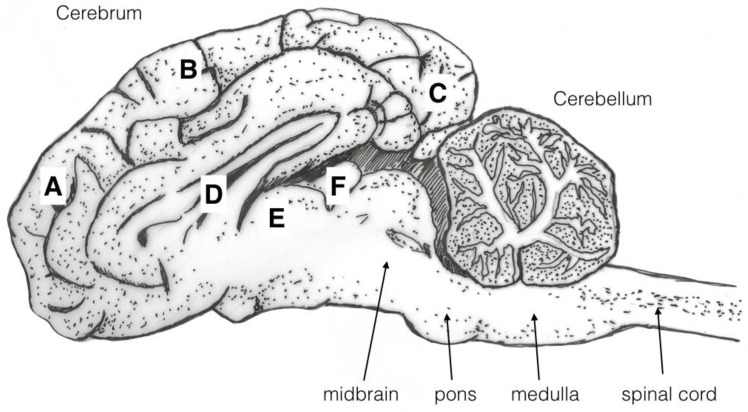
Sagittal diagram of a brain illustrating the areas examined for macroscopic damage. (A) frontal cerebrum, (B) parietal cerebrum, (C) occipital cerebrum, (D) lateral ventricle. These were scored on the basis of 0 = no damage, 1 = slight deformation, 2 = moderate deformation, and 3 = severe deformation of the area. The frontal (A), parietal (B), and occipital cerebrum (C), lateral ventricle (D), thalamus (E), pineal gland (F), midbrain, pons, medulla, and cerebellum were assessed for presence or absence of haemorrhage with a score of 1 indicating presence of haemorrhage and 0 the absence of haemorrhages.

The macroscopic brain lesions were assessed subjectively, and blind to the weight and carcass number, utilising a scale adapted from Sharp et al. [13] and also used in Grist et al. [14] where 0 = no damage, 1 = slight deformation, 2 = moderate deformation, and 3 = severe deformation of the area. The areas examined for macroscopic damage were the frontal, parietal, and occipital cerebrum including the structure of the lateral ventricle and the cerebellum as detailed in Figure 5. This gave a total possible score for damage of 15. The frontal, parietal, and occipital cerebrum, lateral ventricle, thalamus, pineal gland, midbrain, pons, medulla, and cerebellum were also assessed for presence or absence of haemorrhaging with a score of 1 indicating presence of haemorrhage and 0 the absence of haemorrhage, giving a total possible score for brain haemorrhage of 10.

### 2.3. Statistical Analysis

We report simple summary statistics and a 95% confidence interval for a single sample proportion using Wilson’s Method [15], for the effectiveness of the device in producing an effective stun/kill. The joint effects of variables such as carcass weight, and those describing the shot position and damage, on the two outcome variables ‘time to cessation of movement’ and ‘movement score’, were investigated using general linear models (GLM). Predictor variables were simply fitted as main effects and the residuals checked to ensure that the models met the required assumptions. The statistics package IBM Statistics SPSS (v23) was used to produce summary statistics and the GLMs. To facilitate detailed statistical analysis, the shot code for each head was converted to a number to produce a lateral and ventral position score. Using this method, the lateral score L1 became −1, L2 became −2, etc. with right ventral positions being designated as positive scores. The ventral targeting position was converted to a numerical targeting score with A being converted to 1, B = 2, C = 3, etc.

## 3. Results

Every kid (*n* = 200) was effectively stunned, i.e., rendered immediately unconscious. However, 6 of the first 42 kids subsequently demonstrated rhythmic breathing (potentially recovering from the stun) and were humanely killed by an injected overdose of sodium pentobarbitone (Euthatal). Following the review of additional research (M. Sutherland, personal communication, 17 July 2015), the shot position was moved further back between the ears, with the chin tucked into the neck as described by Sutherland et al. [7], and the remaining 158 animals were shot using this revised position. Tucking the chin under the neck allows access to the rear of the head and also stretches the nuchal ligament, reducing the absorption of energy by the latter. Kids numbered 43 to 200 were successfully stun/killed (*n* = 2 developing agonal breathing). With our sample size of 158 kids and with 100% animals effectively stun/killed, this gave a 95% confidence interval of 97.5–100%.

### 3.1. Shot Position

**Figure 6:**
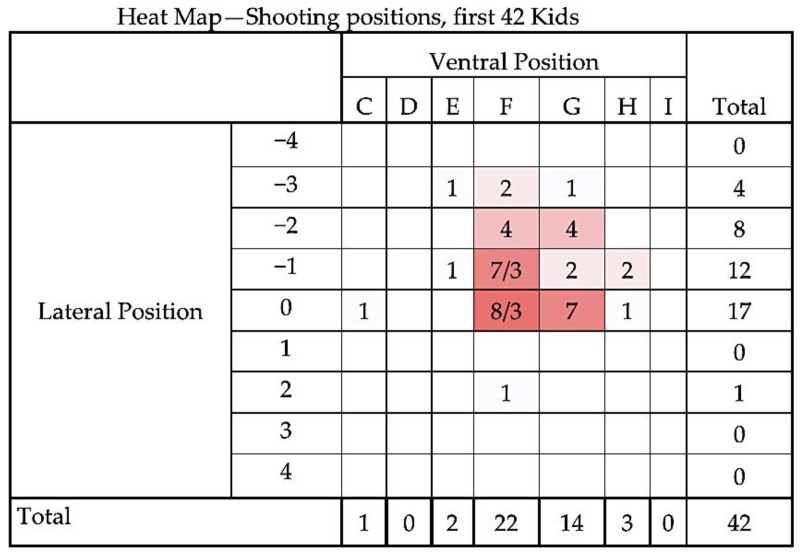
Heat map of the distribution of application positions (right-handed operative) for the first 42 kids. Colour intensity is related to number of applications in each location. Animals euthanised by Euthatal are denoted by highlighted number, for example position −1F was applied to seven animals, of which three required euthanasia due to the presence of rhythmic breathing post application. Lateral target area, with 0 representing the midline based on the suture of the paired frontal bones with positive values to the left hand side of midline and negative values to the right hand side of midline of the head (see Figure 3). Ventral targeting areas using surface topography with F representing a shot on the frontal-parietal suture, G on the parietal bone, H on the parietal-occipital suture etc. with C rostral to the head (see Figure 4).

**Figure 7:**
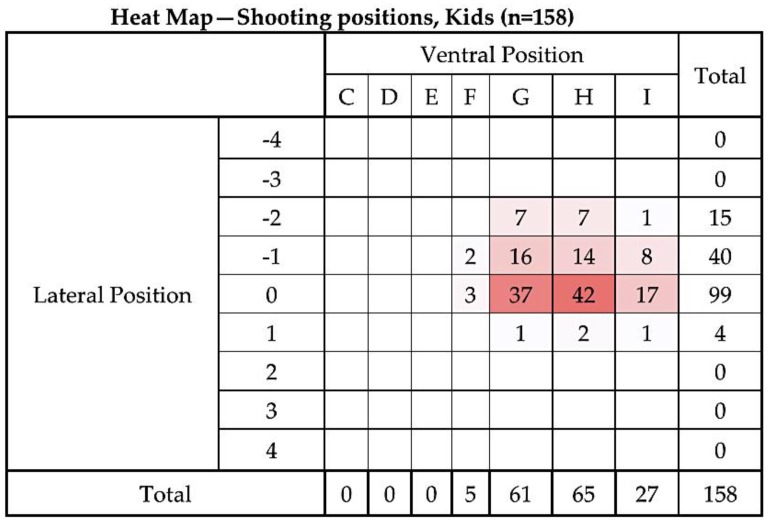
Heat map of the distribution of application positions (right-handed operative) for the remaining 158 kids. Colour intensity is related to number of applications in each location. Lateral target area, with 0 representing the midline based on the suture of the paired frontal bones with positive values to the LHS and negative values to the RHS of the head (see Figure 3). Ventral targeting areas using surface topography with F representing a shot on the frontal–parietal suture, G on the parietal bone, H on the parietal–occipital suture, etc. with C rostral to the head (see Figure 4).

Figure 6 and Figure 7 compare the distribution of application positions for the first 42 kids versus the remaining 158 animals as a heat map, clearly showing the revised and effective shot position. A heat map is a graphical representation of data where the individual values contained in a matrix are represented as colours [16].

**Figure 8:**
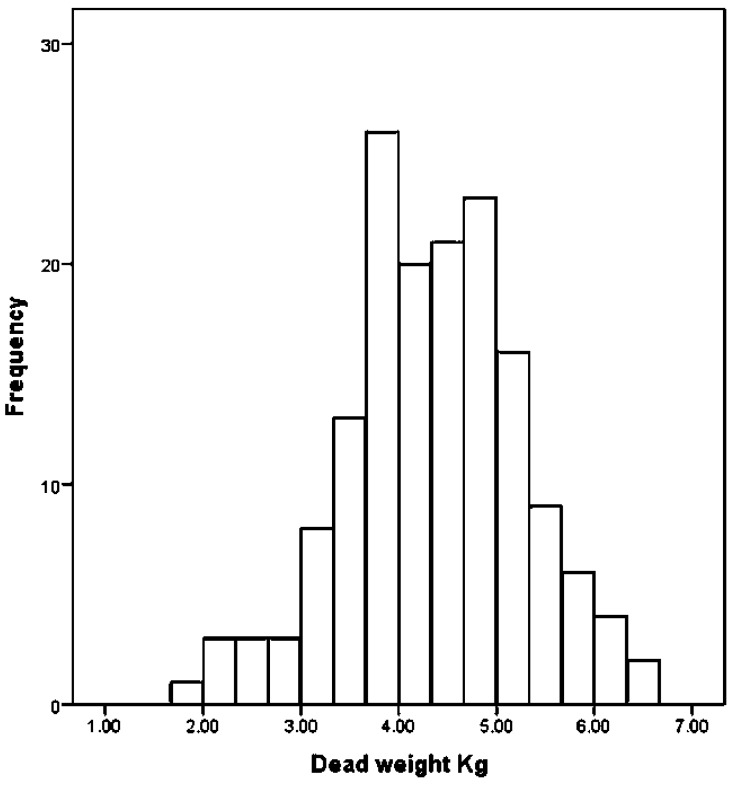
The distribution of kid dead weights (*n* = 158).

**Figure 9:**
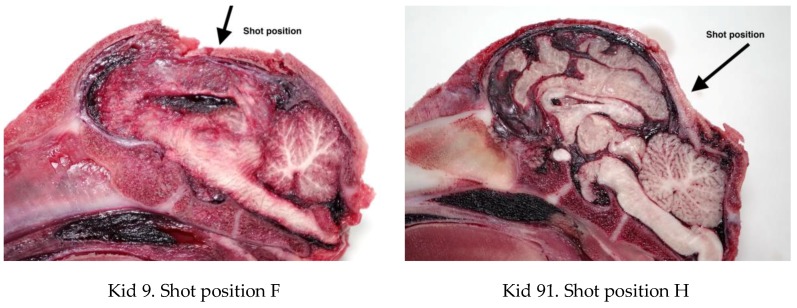
Variation in the damage produced between Kid 9 (forward shot position) and Kid 91 (caudal shot position).

**Figure 10:**
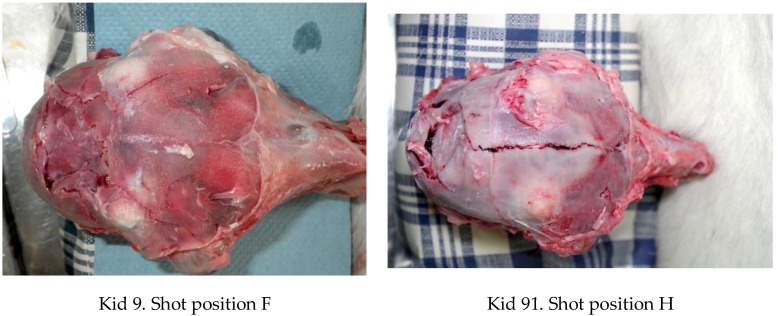
Variation in the fracture pattern produced between Kid 9 (forward shot position) and Kid 91 (caudal shot position).

The first 42 animals have been removed from the statistical analysis presented below and we report only the results for the remaining 158. Figure 8, Figure 9 and Figure 10 describe the distribution of dead weight (kg) mean = 4.35 kg (SD = 0.918), movements scores mean = 2.56 (SD = 0.612) and time to loss of movement (s) mean = 77.39 s (3.037), respectively for the 158 kids.

### 3.2. Factors Related to ‘Time to Loss of Movement’

The parameter estimates from the GLM analysis of ‘time to loss of movement’ are shown in Table 2. This shows that there was no significant effect of dead weight, total damage score, total haemorrhage damage score, or lateral shot position score on the time to loss of movement. There was a significant effect of ventral position score on time to loss of movement with every unit increase in score associated with an increased time to loss of movement of 7.96 s (Table 2).

### 3.3. Factors Related to ‘Movement Score’

The parameter estimates from the GLM analysis of ‘movement score’ are shown in Table 3. This shows that there was no significant effect of total damage score, total haemorrhage score, or lateral shot position score on movement score. There was a significant effect of dead weight on movement score with every 1 kg increase associated with an average increase in movement score of 0.4 (Table 3). There was a weak trend for movement score to be associated with ventral shot position score with, potentially, every 1 unit increase in ventral position score associated with, on average, a 0.1 decrease in movement score (Table 3).

## 4. Discussion

### 4.1. Animal Movement Post Shot

Once the correct shooting position had been established, the Accles and Shelvoke CASH Small Animal Killer (CPK 200) powered by a brown 1 grain cartridge produced an effective and humane stun/kill in all 158 (95% CI = 97.5 to 100%) of neonatal goat kids tested in the study.

When brain dysfunction is induced either by physical trauma, ischaemia, or hypoxia a ‘quiet’ electroencephalogram is present although the carcass can display vigorous convulsions [17]. Post-shot movement is an expected outcome of an effective stun/kill as the brain is no longer suppressing spinal movements [18,19,20]. The degree of post stun movement reflected in the movement scores, recorded during this project, and the time to loss of movement, is an aesthetic concern for stockmen and any bystanders. It is proposed that both the degree and extent of post stun/kill movement will depend on factors such as the physical and nutritional condition of neonates at the time they are killed. The significant effect of ventral shot position on ‘time to loss of movement’ demonstrated that, as the shot position was applied more caudally, the kids displayed movement for longer post shot. It is likely that variation in shot position will affect different areas of the brain and brain stem, which will, in turn, have an effect on spinal reflexes.

The only significant factor, that affected the degree of post-shot movement, was the effect of dead weight where the heavier kids expressed more movement. The larger animals were likely to be better nourished, which could have accounted for an increase in energy available to potentiate post stun movement.

An additional outcome of this project is the production of on-line training material that will instruct stockpersons about restraint, the correct application of the gun including shot position, and the expected post-shot movements.

### 4.2. Shooting Position

Once the optimum shooting position was determined, i.e., a position on the midline, between the ears with the chin tucked into the neck [7] (Kid 91 as shown in Figure 2, Figure 9, and Figure 10) the method was very successful. Initial concerns that a shot placement that was caudal to the parietal-occipital suture would impact the nuchal ligament, which may absorb kinetic energy, and the possibility of shock waves affecting the medulla without affecting the reticular system or ascending reticular activating system (i.e., removing the rhythmic breathing reflex without stunning) [21] were negated by work by Sutherland et al. [7] who demonstrated loss of visual evoked potentials with this shot position, using a lower kinetic energy non-penetrating captive bolt device (27.8 Joules c.f. 47 Joules) (TED, Bock Industries Inc, Philipsburg, PA, USA [8]).

### 4.3. Brain Damage

The caudal shot position (ventral position H, Figure 4) employed with these animals (kids) led to less macroscopic physical damage to the cerebrum when compared with the other species (piglets and lambs) tested [10,11,14].

### 4.4. Fracture Pattern

**Figure 11:**
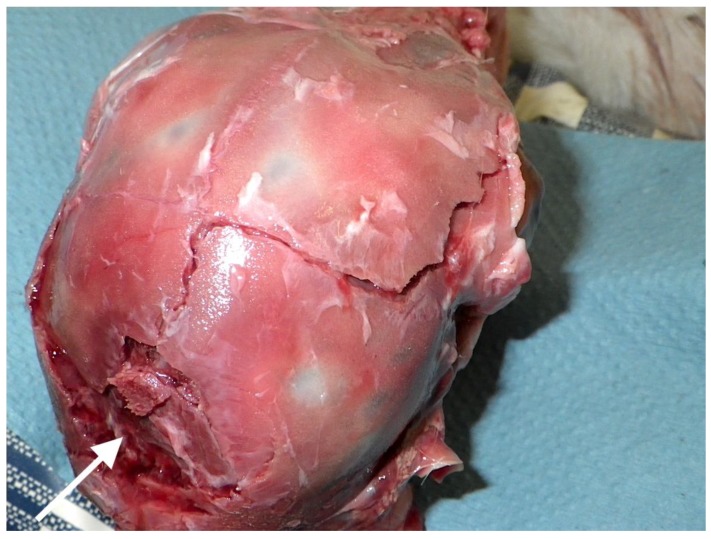
Extension of fracture lines following sk Bock Lane, Philipsburg, PA 16ull sutures Kid 116 (caudal shot position indicated by arrow).

All the heads displayed a depressed fracture corresponding to the impact point, the fracture becoming more discrete with the caudal application adopted (Figure 10 and Figure 11). The caudal application position also resulted in fractures extending from the impact point forward along suture lines (Figure 11).

### 4.5. Agonal Breathing

Of the 158 kids shot in the revised position, 2 animals (1.27%) displayed agonal breathing movements post shot, but no behavioural indicators of brain function. Agonal breathing is quite distinct from the rhythmic breathing associated with perceived consciousness [22], and has been discussed by Grist et al. [10,11,14] and is due to factors separate to those for consciousness; representing a form of residual brain stem activity that ultimately progresses to the death of the animal.

### 4.6. Pronecephaly

**Figure 12:**
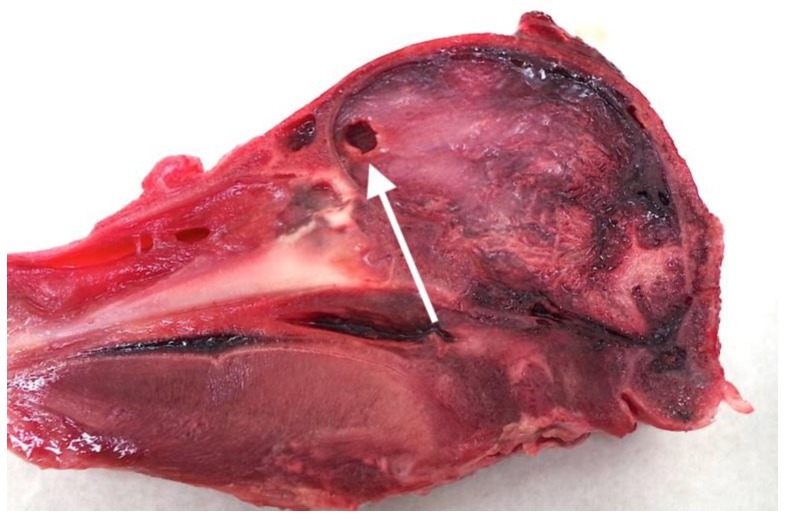
Arrow pointing to a neopallial cyst (pronecephaly) within the frontal cerebrum of Kid 161.

As reported in Grist et al. [10] with neonate piglets, an incidental finding during the post mortem examination of the neonate goat heads was that 10 animals (5%) displayed lesions consistent with abscesses or pronecephaly within the cerebrum, the latter resulting from lack of development and destruction of the cortex, normally due to viral infections in utero [23]. These cysts are usually surrounded by a membrane of astroglial cells that appear white (Figure 12). The fact that 5% of kids presented with pre-existing macroscopic brain lesions should, in the author’s view, be researched further but is outside the scope of this present study.

## 5. Conclusions

It is concluded that the use of the CASH Small Animal Tool (CPK 200), percussive stun/kill device can be recommended for euthanasia of neonate kids when a specific shot position, on the midline, between the ears with the chin tucked into the neck is used in conjunction with a 1 grain cartridge. The shot position is critical and has been confirmed by this work and the work in New Zealand by Sutherland et al. [7]. The previous work demonstrated a loss of VEPs with a weapon delivering 27.8 Joules, the weapon used in this study produced 47 Joules and was successful based on behavioural indicators of brain death.

Provided the correct shot position is employed, the use of this device will improve animal welfare in cases of euthanasia as it allows for reproducible results and will give the stockperson confidence in their ability to reliably produce a single use stun/kill. Post application movement will occur due to the loss of brain control of spinal reflexes and this needs to be explained to the operator.

## Figures and Tables

**Table 1:** Subjective scoring system used to assess post-stun/kill movement based on level of spinal reflex activity, ranging from 0 (no activity post-stun) to 3 (Gross uncontrolled physical movement).

Score	Descriptor	Description
0	No activity	Very little movement
1	Mild activity	Some mild uncontrolled physical movement of limbs
2	Moderate activity	Considerable uncontrolled physical movement of the limbs
3	Severe	Gross uncontrolled physical movement

**Table 2:** Parameter estimates from the GLM testing for an effect of dead weight (kg), total damage score, total haemorrhage score and lateral and ventral shot position scores on time to loss of movement (s).

Parameter	B	S.E.	*t*	Sig.
Intercept	41.695	30.529	1.366	0.174
Dead weight (kg)	−0.052	3.094	−0.017	0.986
Total damage score	−1.296	3.054	−0.424	0.672
Total haemorrhage score	−2.054	2.081	−0.987	0.325
Lateral position score	−1.978	3.895	−0.508	0.612
Ventral position score	7.960	3.648	2.182	0.031

**Table 3:** Parameter estimates from the GLM testing for an effect of dead weight (kg), total damage score, total haemorrhage score, and lateral and ventral shot position scores on movement score.

Parameter	B	S.E.	*t*	Sig.
Intercept	1.447	0.474	3.049	0.003
Dead weight (kg)	0.404	0.048	8.411	≤0.001
Total damage score	−0.015	0.047	−0.313	0.755
Total haemorrhage score	0.008	0.032	0.239	0.811
Lateral position score	0.083	0.061	1.373	0.172
Ventral position score	−0.098	0.057	−1.737	0.084
